# KEAP1 C151 active site catalysis drives electrophilic signaling to upregulate cytoprotective enzyme expression

**DOI:** 10.1016/j.redox.2025.103906

**Published:** 2025-10-28

**Authors:** Matthew R. Schnell, Tianhua Zhai, Edwin R. Ragwan, Hannah Jung, Jiayu Zhang, Anthony F. Lagalante, Yan Kung, Daniel A. Kraut, Zuyi Huang, Aimee L. Eggler

**Affiliations:** a Department of Chemistry and Biochemistry, Villanova University, Villanova, PA, 19085, USA; b Department of Biochemistry and Biophysics, University of Pennsylvania, Philadelphia, PA, 19104, USA; c Department of Biostatistics, Epidemiology and Informatics, University of Pennsylvania Perelman School of Medicine, Philadelphia, PA, 19104, USA; d Department of Chemistry, Bryn Mawr College, 19010, USA; e Department of Chemical and Biological Engineering, Villanova University, Villanova, PA, 19085, USA

**Keywords:** Sulforaphane, Omaveloxolone, Electrophile signaling, Cysteine sensor, KEAP1, NRF2

## Abstract

Cells mount a detoxification, antioxidant, and anti-inflammatory response to electrophiles, mediated by the NRF2 transcription factor. Electrophilic NRF2 activators are used to treat diverse chronic diseases. While the majority of NRF2 activators target C151 of KEAP1, the primary NRF2 repressor, it is unknown how diverse electrophiles favor this particular cellular cysteine. One hypothesis is that the p*K*_a_ of C151 is lowered by surrounding basic residues, resulting in a higher population of the reactive thiolate. We show that the p*K*_a_ of C151 is 6.9, providing optimal reactivity at physiological pH, using the fluorogenic, thiol-reactive electrophile monobromobimane. Surprisingly, monobromobimane reacts with C151 much faster than with a small-molecule thiolate. NRF2 activators in clinical use and trials (omaveloxolone, bardoxolone methyl, and sulforaphane) readily compete with monobromobimane for C151. A BTB-monobimane crystal structure shows no specific orientation in the active site after covalent addition. A 4D flexible BTB model based on seven crystal structures was used to dock mBBr and NRF2 activators into the active site to obtain poses of the pre-covalent enzyme-substrate complexes. They reveal an active site around C151 that accommodates structurally diverse activators using largely hydrophobic interactions, with a hydrogen bond orienting their electrophilic carbons within ∼3–5 Å of C151 for a catalytic proximity effect. In addition, our biochemical and docking results suggest a critical catalytic role for hydrogen bonding to the oxygen of an α,β unsaturated carbonyl, which could both increase carbon electrophilicity and stabilize the negative charge in the transition state. Overall, this work suggests enzymatic catalysis is the primary reason that C151 acts as a sensor cysteine for therapeutic, electrophilic NRF2 activators, highly favoring reaction with C151 over other cellular cysteines. The kinetic targeting of electrophiles such as sulforaphane and omaveloxolone to the C151 active site provides an explanation for how electrophilic compounds can be selective pharmacological agents.

## 1 Introduction

**Fig. 1:**
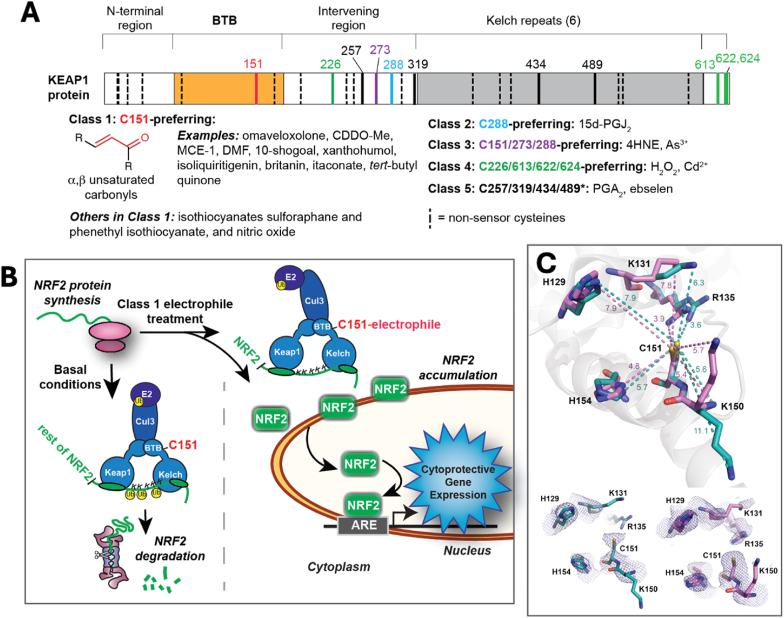
Schematics demonstrating (A) C151 is a preferred KEAP1 Cys target of NRF2 activators, (B) the mechanism of NRF2 regulation by Class 1 electrophiles, and (C) the current hypothesis for C151 selectivity: low p*K*_a_ dictated by surrounding basic r**esidues. (***A*) The KEAP1 domains in the primary sequence, with the locations of the 27 cysteines, are indicated. The five classes of cysteine-reactive NRF2 activators are shown as defined by the Yamamoto group [5], along with members of Classes 2–5 as described in that work. ∗Class 5 activators are dependent on one or more of the four cysteines indicated, with their specific targets among those four yet to be determined [5]. *(B)* A primary mechanism by which NRF2 is regulated. NRF2 is constantly synthesized and quickly targeted for degradation by ubiquitination. KEAP1 forms a bridge between NRF2 and the CUL3 protein, part of an E2 ubiquitination complex. Class 1 electrophiles free NRF2 from KEAP1 suppression by modifying C151 and causing a conformational change that prevents ubiquitination of NRF2 lysines. *(C)* PDB 4cxi (in teal) and 7exi (in purple), showing the distances between C151 and the surrounding basic residues (upper panel) and the lack of defined electron density for the lysines and arginine (lower panels), indicating flexibility in these residues.

A wide variety of endogenous and exogenous electrophiles trigger a coordinated response in cells, in which the NRF2 transcription factor is activated to upregulate the expression of enzymes to neutralize and eliminate electrophiles. In addition to preventing the deleterious reaction of these electrophiles with cellular components, NRF2 also upregulates genes with repair, antioxidant, and anti-inflammatory functions. Given that oxidative stress and inflammation underlie most chronic diseases, including heart disease, cancer, neurodegenerative diseases and diabetes, significant effort has been invested over three decades to understand and harness this response to combat and prevent toxic exposures and chronic diseases. Three small molecules in current clinical use for this purpose are themselves electrophiles: dimethyl fumarate (DMF, Tecfidera) to treat multiple sclerosis [1]; omaveloxolone, the first FDA-approved molecule designed for this purpose, currently for treatment of Friedrich's Ataxia [2]; and sulforaphane sourced from broccoli sprouts, the subject over 90 clinical trials for a broad array of chronic diseases including those listed above [3,4]. A central question in the development of potent and low-toxicity NRF2 activators is how the cell senses such a structurally diverse array of electrophiles, signaling to upregulate NRF2, before the electrophiles react with the many other potential targets in the cell, including glutathione. A major step forward was the discovery in 1999 that the primary repressor of NRF2 is KEAP1, with 27 cysteines in humans (Fig. 1A), 25 of which are highly conserved [6]. The cysteine residue thiol, when deprotonated, is a soft nucleophile and thus a preferred target for the typical NRF2 activator, which is a soft electrophile. Extensive efforts in the field since then [[7], [8], [9]], culminating with a comprehensive investigation by the Yamamoto group using transgenic mice-derived cell lines with mutation of these cysteines [10,11], have determined that 11 of these cysteines are required for sensing and responding to known NRF2 activators, with five defined classes as of 2020 [5] (Fig. 1A).

The vast majority of NRF2 activators that have been assessed for KEAP1 cysteine-dependence are in Class 1 (Fig. 1A), meaning C151 is required for activity at lower and generally physiologically relevant concentrations of the activator [12,13]. Mass spectrometry studies of full-length KEAP1 with its 27 cysteines confirm that C151 is the most readily-modified cysteine *in vitro* by Class 1 molecules (reviewed in Naidu et al.) [14], indicating that they are particularly reactive with C151. In general, C151 is required for KEAP1/NRF2 to sense almost all known α,β unsaturated carbonyl NRF2 activators as well as the isothiocyanates sulforaphane [9,10,15] and phenethyl isothiocyanate (PEITC) [12] (Fig. 1a), which are all soft electrophiles [16]. Examples of Class 1 α,β unsaturated carbonyls include omaveloxolone [17] and the closely-structurally related 2-cyano-3,12-dioxooleana-1,9(11)-dien-28-oic acid methyl ester (CDDO-Me) [18] and CDDO-imidazole (CDDO-Im) [10], all highly potent semi-synthetic derivatives of oleanolic acid tested in clinical trials, and the much simpler but still highly potent cyanoenone, 3-ethynyl-3-methyl-6-oxocyclohexa-1,4-dienecarbonitrile (MCE-1) [18]. This class also includes DMF [19], and a kinetic study of DMF with KEAP1 shows a higher rate of reactivity of DMF with C151 at pH 7.5 than other KEAP1 cysteines or glutathione [20]. Given that the majority of natural product electrophilic NRF2 activators are α,β unsaturated carbonyls or are easily bio-converted to α,β unsaturated carbonyls [16], many NRF2 activators from the diet and other natural sources also target C151 [14,16], including xanthohumol from hops, isoliquiritigenin from licorice, and 10-shogaol from ginger [15], as well as britanin from the genus Inula, used in traditional Chinese medicine [21]. Endogenous Class 1 α,β unsaturated carbonyls include the Krebs cycle-derived metabolite itaconate [22], with implications in particular for the metabolite's anti-inflammatory responses (reviewed in Ref. [23]). The endogenous signaling molecule nitric oxide also is C151-dependent [8]. Conjugation of a Class 1 electrophile with KEAP1 C151 inactivates KEAP1's ability to target NRF2 for ubiquitination, resulting in rapid NRF2 accumulation (Fig. 1B). After translocation to the nucleus, NRF2 binds to copies of its cognate response element, known either as the electrophile response element or the antioxidant response element, upregulating gene expression.

How are the structurally diverse Class 1 electrophiles targeted specifically to KEAP1 C151, evading millimolar concentrations of glutathione and cellular protein thiols? Beyond the common α,β unsaturated carbonyl moiety, these molecules share no other structural features. A leading hypothesis in the field [5,8,14] is that nearby basic residues H129, K131, R135, K150, and H154 lower the p*K*_a_ of C151 (Fig. 1C), stabilizing the thiolate (deprotonated) form at physiological pH and thus increasing its reactivity with electrophiles, since only the thiolate and not the thiol form can react with an electrophilic carbon. In support of this hypothesis, the triple mutant KEAP1 K131 M/R135 M/K150 M behaved like KEAP1 C151S, repressing NRF2 in cells and responding to non-Class 1 NRF2 activators such as Cd^2+^ but not to sulforaphane [8]. The p*K*_a_ of C151 has not yet been determined. It is also unclear if the p*K*_a_ is the sole source of C151's reactivity, or whether the residues surrounding C151 play additional roles in targeting Class 1 electrophiles to this specific cysteine. Our initial goal in this work was to determine the p*K*_a_ of KEAP1 C151. As described herein, the thiolate-reactive probe monobromobimane (mBBr) used to determine the p*K*_a_ revealed much more about the chemistry driving the specificity of Class 1 electrophiles with C151.

## 2 Results

### 2.1 The p*K*_a_ of KEAP1 C151 is optimized for reactivity at physiological pH; the probe mBBr, used to determine the p*K*_a_, reacts remarkably quickly with C151

**Fig. 2:**
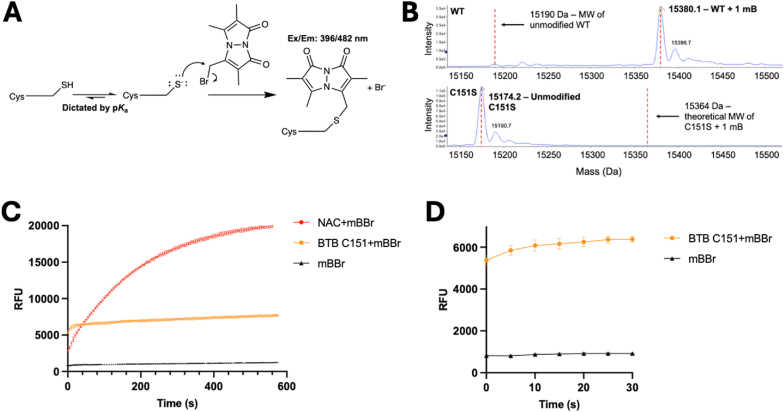
mBBr rapidly reacts with KEAP1 BTB C151 compared with *N*-acetyl c**ysteine. (***A*) A schematic illustrating the use of mBBr to determine the p*K*_a_ of a cysteine. Only the deprotonated, thiolate form reacts with electrophiles. The conjugate product of monobimane with a cysteine is detected by fluorescence. (*B*) Top-down mass spectrometry of the WT BTB protein (top) and BTB C151S protein (bottom) (1.6 μM) reacted with mBBr (16 μM) for 1 h at room temperature. (*C*) The reaction at 25 °C of 16 μM mBBr with 1.6 μM of either NAC at pH 11 or BTB C151 at pH 8 was monitored over time, with relative fluorescence units (RFU) monitored as shown in A. (*D*) Early time points from *C*.

To determine the p*K*_a_ of KEAP1 C151, we used a construct containing only three cysteines (versus 27 in full-length KEAP1) consisting of just the BTB (Broad-Complex, Tramtrack and Bric-a-brac) domain (residues 48–180, with a S172A mutation), which has been widely employed for crystallographic studies of C151-targeted electrophiles (*e.g.*, Ref. [24]). To assess the protonation state of C151 at various pHs, the thiolate-reactive probe monobromobimane (mBBr) was used, as it produces a fluorescent monobimane-thiol conjugate (Fig. 2A) [25]. While mBBr is primarily used as a derivatizing agent to quantitate biological thiols [[25], [26], [27]], it has been used to determine protein cysteine p*K*_a_s, for example by measuring the initial rate of reaction of the probe with human peroxiredoxin 5 as a function of pH [28]. In addition, its high sensitivity to the polarity of its molecular environment has enabled its use as a probe for protein conformational changes [29,30]. mBBr is considered a “naive” reagent that should react with all thiols at a similar rate, given no enzymes other than glutathione *S*-transferases are known to use it as a substrate or specific ligand [28,31].

To determine whether mBBr reacts only with C151 in the BTB domain, and not the other two thiols present (C77 and C171), which are fairly buried in the crystal structures of this domain, top-down mass spectrometry was performed after incubating the BTB protein with a 10-fold excess of mBBr for 1 h. As shown in Fig. 2B, the BTB protein was completely modified by one monobimane (no peak was observed at the *m*/*z* for two modifications, not shown). In contrast, for the C151S BTB protein, the protein was unmodified by monobimane, confirming that only C151 is modified in WT BTB. Next, the change in fluorescence of the reaction of a 10-fold excess of mBBr with the simple thiol *N*-acetyl cysteine (NAC) was monitored over time at pH 11, ensuring that the NAC cysteine, p*K*_a_ of 9.43, was deprotonated (Fig. 2C). Because mBBr is photosensitive, reads were taken every 5 s, preventing interference from photoreaction (*SI Appendix,* Fig. S1). As expected, the fluorescence increased until the reaction was complete, in approximately 10 min. Unexpectedly, when mBBr was incubated with the BTB domain protein at pH 8, the fluorescence rapidly increased with the first read, and only slightly increased over the next 20 s, remaining stable for the time it was monitored (10 min) (Fig. 2C and D). In addition, the RFU signal was considerably lower for the BTB-C151 monobimane conjugate than for the NAC-C151 conjugate, despite reaction conditions being identical to those in Fig. 2B, showing a complete reaction of C151 with mBBr. These results indicated that the environment surrounding C151 had two effects on the reaction: first, to significantly increase the reaction rate, and second, to suppress the conjugated monobimane fluorescence quantum yield, as has been observed previously for the monobimane-thiol conjugates in hydrophobic protein environments [29,30].

**Fig. 3:**
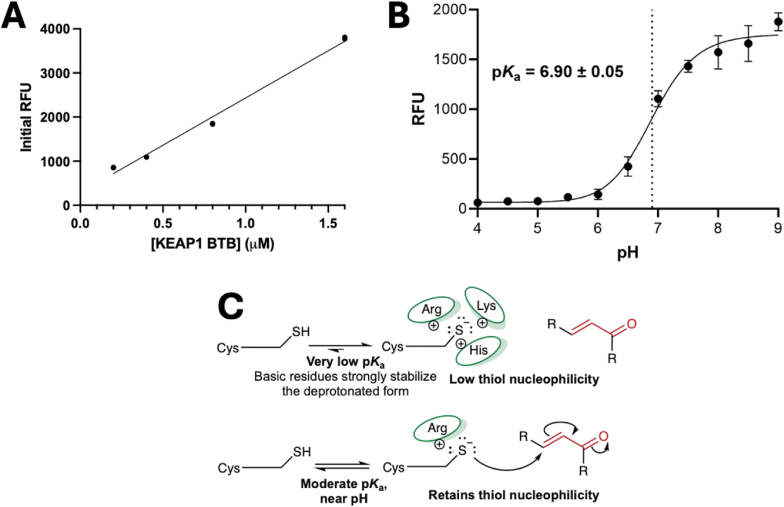
Determining the p*K*_a_ of C151 by reaction **with mBBr.** (*A*) The initial fluorescence of the reaction of 16 μM mBBr with various concentrations of BTB C151 at pH 8 was determined. (*B*) At the same conditions as in *A*, 0.8 μM WT BTB protein was reacted with mBBr and initial fluorescence was determined. The RFU of mBBr alone was subtracted, and the data were fit to obtain the p*K*_a_. (*C*) A schematic illustrating that a very low p*K*_a_, relative to the reaction pH, blocks the reactivity of a cysteine by stabilizing the thiolate to an extent that reduces its nucleophilicity. In contrast, a moderate p*K*_a_, near the pH, balances deprotonation of the thiol to the thiolate with maintenance of the nucleophilicity of the thiolate.

To determine the p*K*_a_ of KEAP1 C151, we took advantage of the rapid reaction of mBBr with C151 and predicted that the initial RFU (at the first timepoint taken) would correlate with the amount of free thiolate. To test this, increasing concentrations of the BTB domain protein, and thus increasing concentrations of the BTB thiolate, were reacted with excess mBBr, and a linear correlation was observed from 0.2 to 1.6 μM BTB (Fig. 3A). Incubation of BTB with excess mBBr at pH values from 4 to 9 (Fig. 3B) resulted in an initial RFU versus pH plot that fit well to the Henderson-Hasselbach equation, with a p*K*_a_ of 6.90 ± 0.05. As expected, the protein environment around C151 lowered its p*K*_a_ from 8.6 for free cysteine in aqueous solution [32].

### 2.2 The use of x-ray crystallography and 4D molecular modeling to visualize the interactions of mBBr with the BTB-C151 active site

While a p*K*_a_ of 6.9 does increase the amount of C151 thiolate available to react with a Class 1 electrophile, and potentially explains selectivity for C151 over other KEAP1 cysteines, a lower p*K*_a_ does not fully explain the ability of a Class 1 electrophile to react with KEAP1 C151, present at ∼1 μM in the cytoplasm [33], compared to the much higher concentration of other cellular thiols. The rapid reaction of monobromobimane with KEAP1 C151 compared to NAC (Fig. 2C and D) indicates that residues surrounding C151 significantly enhance the rate of reactivity of C151 with monobromobimane compared to that of a simple cysteine such as NAC or reduced glutathione (GSH). A better understanding of how the reaction between C151 and mBBr is “catalyzed” may help in understanding how Class 1 NRF2 activators are targeted to C151. (We use the term catalysis here in the context of increasing the rate of reaction, but not for the regeneration of the enzyme, akin to a covalent mechanism-based inactivator of an enzyme.)

**Fig. 4:**
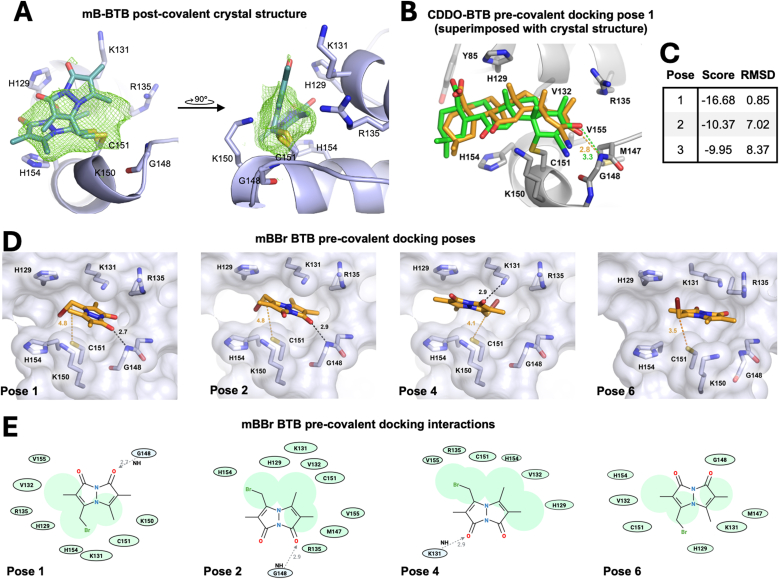
Visualizing the interactions driving mBBr reaction catalysis by the C151 pocket. A 1.80 Å crystal structure of the BTB modified at C151 with monobimane in two alternate conformations whose occupancies refined to 0.48 and 0.52. Polder *mF*_*o*_*–DF*_*c*_ omit density [34] for monobimane is contoured at 2.0 σ (green mesh). (*B*) A 4D flexible model of the BTB domain containing the C151 pocket was used to dock CDDO, and the resulting top scoring pose (in yellow) was overlaid with the crystal structure of CDDO bound in the pocket (PDB 4cxt, in green). (*C*) The top three scoring poses from the docking of CDDO in a pre-covalent manner to the C151 pocket using the 4D model, where the top pose has a distinctly larger negative value than the next two highest scores and has a distinctly lower RMSD for its position overlaid with the crystal structure ligand position. (*D*) The four poses for the pre-covalent docking of mBBr to the C151 pocket. See text for details. The distance from the electrophilic carbon to C151 is indicated in yellow, and hydrogen-bonds and their distance are indicated in black. (*E*) The hydrophobic (green) and hydrogen bond (light blue) interaction maps corresponding to the poses in *D*. The relative contribution of a hydrophobic interaction for a given residue corresponds to the size of the residue oval.

As a first step to visualize how the reaction of C151 with mBBr is catalyzed, the BTB-bimane conjugate (verified by mass spectrometry as in Fig. 2B) was crystallized and diffracted to 1.80 Å resolution (Fig. 4A, *SI Appendix*, Table S1). Positive electron density near C151 indicated that ligand was bound. However, the density was ambiguous and not continuous, suggesting that the ligand was flexibly bound and adopting multiple conformations. Moreover, the covalently-bound monobimane adduct could bind quite differently from its precursor monobromobimane in a pre-covalent docking interaction, “ES”, which is the interaction of interest to understand the rapid rate of reaction.

To visualize how a pocket around C151 might catalyze the reaction of mBBr with C151, a 4D molecular docking approach was employed. In the available crystal structures of the BTB domain, the residues around C151 adopt a wide variety of conformations (*e.g.*, apo structures PDB 4cxi and 7exi, Fig. 1C). The rotation of residues significantly changes the size and shape of the binding pocket, thereby highly impacting docking results obtained with the traditional Internal Coordinate Mechanics (ICM) method, which docks a flexible small-molecule ligand to a rigid, known receptor 3D structure. A 4D flexible protein receptor that incorporates multiple crystal structures increases the number of sample binding poses to avoid missing likely candidates [35]. ICM-pro was used to create a flexible protein model using seven structures of the KEAP1 BTB domain covalently bound to various Class 1 electrophiles. A ligand-binding pocket around C151 was found, and a docking box was generated 5 Å around the pocket (*SI Appendix*, Fig. S2). The ability of the program to accurately dock the Class 1 electrophile CDDO in a pre-covalent ES complex was assessed (Fig. 4B–C). The top-scoring pose (“Pose 1”) showed by far the best overlap with the crystal structure of CDDO bound to C151 (Fig. 4B). Pose 1 has a distinctly larger negative docking score than the other poses and a much lower RMSD for its position overlaid with the crystal structure ligand position (Fig. 4C). The distinctly higher negative score of Pose 1 and the expected positioning gives confidence in the ability of the 4D model (which was generated without using any ligand information) to obtain a reasonable estimation of the interactions that drive the pre-covalent binding position in the active site. Next, mBBr was similarly docked using the 4D ICM method. In contrast to CDDO, the top ten scoring poses did not show a single score that was distinctly more negative than the others (*SI Appendix*, Table S2). Upon examination of the top ten poses, only four (Fig. 4D) were located within the pocket, noting that the docking box extends well beyond the C151 pocket (*SI Appendix*, Fig. S2). For each of these four, the distance between the electrophilic carbon and C151's thiol sulfur is 5 Å or less, which could help explain how the ligand-binding pocket facilitates the reaction. Three of the poses utilize a hydrogen bond, and a number of hydrophobic interactions were identified for each pose (Fig. 4E and *SI Appendix*, Table S3). Overall, the data suggest that monobromobimane, with its relatively flat geometry, largely hydrophobic nature, and possibly its ability to make hydrogen bonds, fits well into the relatively flat, hydrophobic C151 pocket in multiple orientations that place its electrophilic carbon near the C151 thiolate, significantly accelerating the reaction relative to the simple thiolate in NAC. C151-conjugated monobimane was also docked using this method (*SI Appendix*, Table S2). The scores overall were lower than for the pre-covalent docking, and again, no one pose had a distinctly larger and more negative docking score than the others, supporting our hypothesis that the lack of clear electron density for monobimane in the crystal structure is the result of the ligand adopting multiple conformations.

### 2.3 The BTB-C151 active site catalyzes the reaction of C151 with class 1 electrophiles

**Fig. 5:**
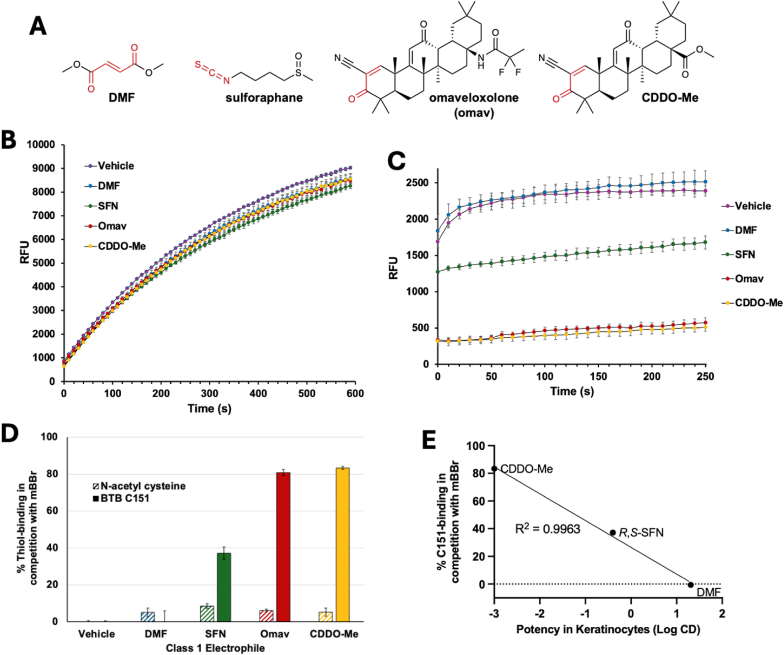
The KEAP1 C151 active site catalyzes the reaction with Class 1 electrophiles. (*A*) Structures of the four Class 1 electrophiles tested in this work, with the soft electrophilic moieties that react with C151 shown in red. (*B*) A “head-to-head” competition between NAC and each of the four Class 1 electrophiles. (“SFN” is sulforaphane and “Omav” is omaveloxolone.) Equimolar amounts of mBBr and each electrophile were added to NAC (8 μM and 0.8 μM final concentrations), and the fluorescence was monitored. (*C*) A “head-to-head” competition for KEAP1 BTB C151 between mBBr and each of the four Class 1 electrophiles. Equimolar amounts of mBBr and each electrophile were added to BTB (8 μM and 0.8 μM final concentrations), and the fluorescence was monitored. (*D*) Quantitation of the percent of thiolate that a given electrophile can bind in a head-to-head competition with mBBr, as described in the Methods. (*E*) Results from *D* were plotted versus the potency of each Class 1 electrophile in HaCaT keratinocytes [36], determined as the log of the concentration required to double reporter activity (CD) value, with the most potent electrophile having the most negative value.

The ability of the KEAP1 C151 active site to catalyze the addition of mBBr to C151 suggests a mechanism by which Class 1 electrophiles can target this particular cysteine in the cellular milieu, inducing NRF2 activation at low μM or nM concentrations. The residues surrounding C151 may function as an active site, catalyzing the addition of Class 1 electrophiles to C151. DMF was recently shown to readily react with protein cysteine residues, in particular KEAP1 C151 [20]. The KEAP1 C151S full-length protein had a 10-fold lower *k*_2_ than did WT KEAP1, showing kinetic targeting of DMF to this cysteine compared to the others in KEAP1. We hypothesized that Class 1 electrophiles, which are generally expected to have weak electrophilic character compared to mBBr, would have little to no ability to compete with mBBr for a simple thiolate such as *N*-acetyl cysteine, but perhaps they would be able to compete with mBBr for KEAP1 C151, depending on whether the residues surrounding C151 acted as an active site to catalyze addition. To test this hypothesis, four Class 1 electrophiles were selected (Fig. 5A): DMF, sulforaphane, omaveloxolone, and CDDO-Me. The compounds were each mixed with an equimolar concentration of mBBr and added to reactions containing *N*-acetyl cysteine. As shown in Fig. 5B–D, as expected, each compound only slightly blocked mBBr from reacting with the thiolate. When the compounds mixed with equimolar mBBr were added to the BTB domain protein, sulforaphane, omaveloxolone and CDDO-Me were all able to block mBBr from reacting with C151 to a significant extent (Fig. 5C–D). These three Class 1 electrophiles thus all react with C151 at a rate that is similar to or faster than the already very fast reaction of mBBr. Their relative inability to compete with mBBr for *N*-acetyl cysteine supports the hypothesis that the residues surrounding C151 act as an active site that is particularly well suited to catalyze the reaction for these particular compounds. In addition, the abilities of CDDO-Me, sulforaphane, and DMF to react with C151 correlate with their potency to induce NRF2-driven gene expression, as shown when comparing the ability to block mBBr binding to the activity of a luciferase reporter in HaCaT keratinocytes as determined by Copple et al. [36]. In sum, the data support the hypothesis that an active site surrounding C151 catalyzes its reaction with Class 1 electrophiles, and electrophiles that are the best substrates for this site are among the most potent known inducers.

### 2.4 KEAP1-C151 active site interactions with class 1 electrophiles

**Fig. 6:**
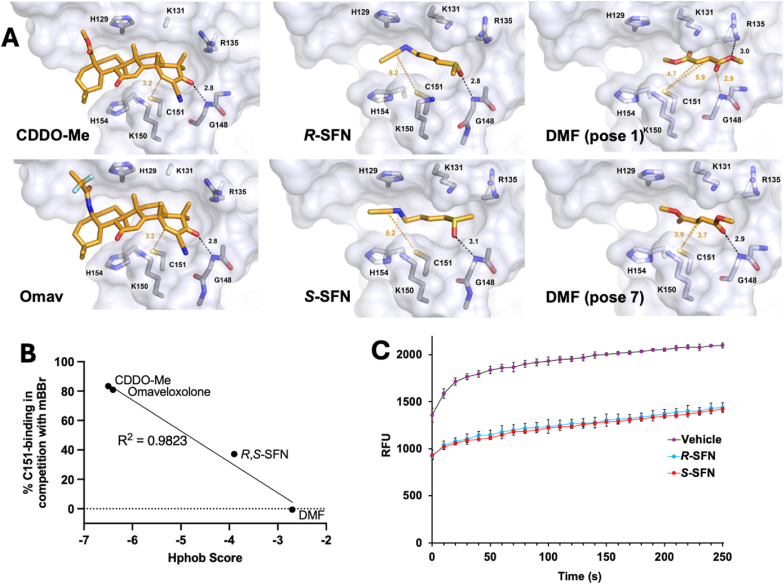
Visualizing the interactions driving Class 1 electrophile reaction catalysis by the C151 pocket. (*A*) The top-scoring poses for the pre-covalent docking of each electrophile to the C151 pocket. See text for details. The distance from the electrophilic carbon to C151 is indicated in yellow, and hydrogen-bonds and their distance are indicated in black. (*B*) The percentage of C151 thiolate that a given electrophile can bind in a head-to-head competition with mBBr plotted versus the hydrophobicity score from the docking. (*C*) A “head-to-head” competition for KEAP1 BTB C151 between mBBr and either *R*–SFN or *S*-SFN. Equimolar amounts of mBBr and each electrophile were added to BTB (8 μM and 0.8 μM final concentrations), and the fluorescence was monitored.

The 4D-flexible docking model provides a means to visualize what interactions in the active site might be driving catalysis. The top-scoring docked structures (*SI Appendix*, Table S4) for CDDO-Me and omaveloxolone (Fig. 6A) closely resemble those of CDDO in the docked structure and the crystal structure (Fig. 4B), including the hydrogen bond between the α,β unsaturated carbonyl oxygen and the backbone nitrogen of G148. The distance from the C151 sulfur atom to the electrophilic carbon, 3.2 Å, suggests the catalysis is largely driven by the proximity effect, that is, a high effective concentration of the reactive groups in combination with restricted translational and rotational motions, one of the largest contributors to enzyme catalytic efficiency [37,38]. Numerous hydrophobic interactions participate in docking (*SI Appendix*, Table S5), with eleven active site residues for CDDO-Me for a hydrophobic bonding score of −6.4 kcal/mol (*SI Appendix*, Table S4) and an additional residue for omaveloxolone for twelve residues total and a hydrophobic bonding score of −6.4 kcal/mol. For sulforaphane, for which a crystal structure bound to C151 is lacking, both stereoisomers were docked. Interestingly, the top scoring pose for each isomer also showed a single hydrogen bond from its sulfoxide oxygen to the backbone nitrogen of G148 (Fig. 4A). Various hydrophobic interactions (*SI Appendix*, Table S6) with nearly equivalent hydrophobic bonding scores of −3.8 and −3.9 kcal/mol were found for *R*- and *S*-SFN, respectively (*SI Appendix*, Table S4). The orientation places the electrophilic carbon for each isomer at 5.2 Å from C151's sulfur, within the typical range of distances relevant for the proximity effect (3–6 Å).

For DMF, the weakest of the tested Class 1 NRF2 activators both in terms of its potency in HaCaT cells and its ability to compete with mBBr, the difference in docking scores between the top-scoring pose and the other determined poses is not nearly as distinct as for the other electrophiles (*SI Appendix*, Table S4). Of the top ten poses, only poses 1 and 7 fulfill the criteria of being located within the C151 pocket. In addition, these are the only two that form a hydrogen bond (Fig. 6A). For pose 7, the hydrogen bond is formed between an α,β unsaturated carbonyl oxygen and the backbone nitrogen of G148, and for pose 1, between the ester oxygen and a nitrogen atom on R135. We note that in pose 1, the α,β unsaturated carbonyl oxygen is 2.9 Å from the nitrogen of G148, though ICM docking did not include this as an interaction. The two electrophilic carbons in DMF are both within distances of C151 relevant for a proximity effect in both poses, and the hydrophobic interaction scores for the two poses are very similar, −2.6 and −2.8 kcal/mol, indicating that either one could be a viable representation of how the C151 active site may interact with DMF to catalyze the reaction with C151.

While a top-scoring docking pose is considered a good indicator of which pose most likely reflects pre-covalent binding interactions, the absolute docking score is generally not a useful indicator of experimental reactivity or potency [39]. Accordingly, as shown in *SI Appendix*, Fig. S4, there is no correlation of docking scores with either the percentage of C151 thiolate that a given electrophile can bind in a head-to-head competition with mBBr (% C151 blocking) or its potency in keratinocytes (data from Fig. 5D). However, plotting % C151 blocking versus the hydrophobic interaction score from docking shows a remarkably linear correlation (Fig. 6B), and by extension from Fig. 5E, the hydrophobic interaction scores correlate well with potency in keratinocytes. (For ease of display, the nearly equivalent hydrophobic scores for *R*- and *S*-SFN were averaged, as were the nearly equivalent scores for poses 1 and 7 for DMF.)

Together, the docking results, the mBBr competition results, and the p*K*_a_ of C151 result support a model in which the C151 active site catalyzes reactions by optimizing the p*K*_a_ to 6.9, by lining the active site with at least 12 residues capable of making hydrophobic interactions, and by orienting the electrophilic carbon within ∼3–5 Å of C151 for a proximity effect. As a test of this model, the combination of nearly equivalent hydrophobic interaction scores for *R*- and *S*-SFN and the hydrogen bond that places each electrophilic carbon at the same distance from the C151 thiolate suggests that they should have a similar ability to block mBBr from reacting with C151 in the head-to-head competition assay. The stereoisomers were individually tested in the same assay used in Fig. 5C to test the racemic mixture. As shown in Fig. 6C, they were equally able to compete with mBBr, indicating that they have very similar rates of reaction with C151.

**Fig. 7:**
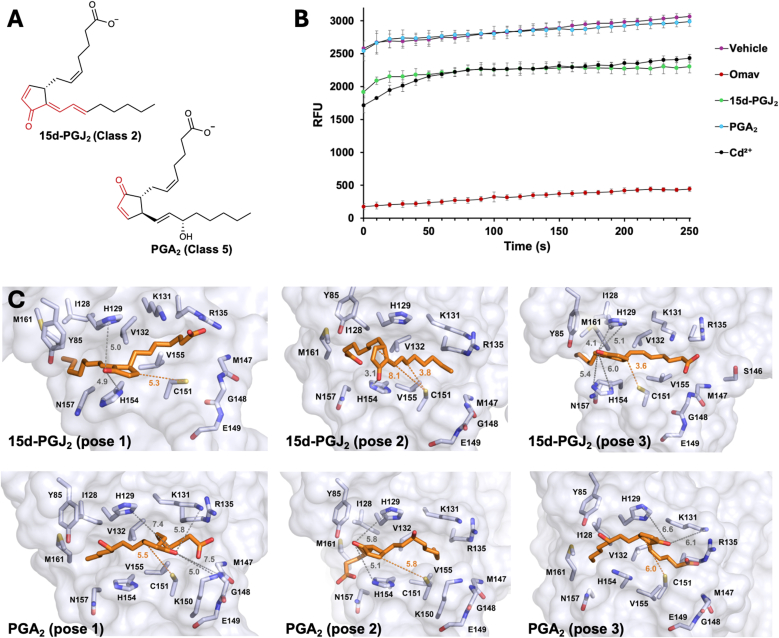
Class 2, 4 and 5 NRF2 activators are relatively weak substrates for the C151 pocket. (*A*) Structures of the Class 2 and Class 5 electrophiles tested, 15d-PGJ_2_ and PGA_2_, with the soft electrophilic moieties shown in red. (*B*) A “head-to-head” competition for KEAP1 BTB C151 between mBBr and each of the thiol-reactive molecules (Cd^2+^ is from Class 4, and Omav is a positive control). Equimolar amounts of mBBr and each electrophile were added to BTB (8 μM and 0.8 μM final concentrations), and the fluorescence was monitored. (*C*) The three top-scoring poses for the pre-covalent docking of 15d-PGJ_2_ and PGA_2_ to the C151 pocket. The distance from the electrophilic carbon(s) to C151 is(are) indicated in yellow, and distances between the carbonyl and the nearest candidates for hydrogen bonding are indicated in gray.

To better understand how the C151 active site catalyzes reactions, we investigated NRF2 activators from Classes 2, 4 and 5, which have been shown to not target C151, or at least, to favor reaction with other KEAP1 cysteines. Endogenous, prostaglandin-derived 15d-PGJ_2_ and PGA_2_ (Fig. 7A) are from Classes 2 and 5, respectively, and the toxic heavy metal cadmium ion is from Class 3. The hypothesis being tested is that the C151 active site has substrate specificity for Class 1 electrophiles (and nitric oxide), and that thiol-reactive species in Classes 2, 4 and 5 would be weak substrates for the active site. The prediction based on this hypothesis is that NRF2 activators from these classes would be unable to compete with mBBr in a head-to-head competition for C151. Using the same assay as in Figs. 5C and 15d-PGJ_2_, PGA_2_, and CdCl_2_ were each tested for their ability to compete with mBBr. As shown in Fig. 7B, PGA_2_ was unable to compete, consistent with the prediction that this endogenous NRF2 activator targets other cysteines due to the inability of the C151 active site to catalyze the addition. Interestingly, 15d-PGJ_2_ and CdCl_2_ showed some ability to block mBBr binding to C151, suggesting that they could also react with C151 in the cellular environment. The consistency of this result with the literature is detailed in the Discussion section. To understand how the C151 active site might catalyze the reaction with 15d-PGJ_2_ to some extent, but not with PGA_2_, the 4D-flexible docking model was used. The docking scores of the top-scoring docked poses were not distinct from the lower-ranked poses (*SI Appendix*, Table S7), as was the case for the pose scores for the poorly reacting DMF. The top three poses for each compound are shown in Fig. 7C. All fit well within the C151 pocket, and the hydrophobic interaction scores for all six poses are closely clustered, from −5.3 to −6.3 kcal/mol. Only one of the three poses for PGA_2_ (pose 1) places the sole electrophilic carbon within reaction distance (5.5 Å) from the C151 thiolate. However, in pose 1, as well as the other two poses, no residue lies within hydrogen-bonding distance of the carbonyl oxygen, indicating the presence of such a residue may be critical. For 15d-PGJ_2_, all three poses show the C151 thiolate within a reasonable reaction distance of at least one of the electrophilic carbons. In pose 2, H154 is within hydrogen bonding distance (3.1 Å) of the carbonyl oxygen—perhaps explaining the ability of 15d-PGJ_2_ to compete with mBBr. Interestingly, the electrophilic carbon within reaction distance of C151 in pose 2 is the δ-carbon in the alkyl side chain, rather than the β-carbon in the ring. The endocyclic β-carbon is the highly preferred site for glutathione addition and is generally thought to be critical for biological activity [40].

## 3 Discussion

This work identifies an active site surrounding KEAP1 C151 that significantly increases the rate at which the electrophilic carbon of Class 1 NRF2 activators reacts with this sensor cysteine compared to ordinary cysteines. This catalysis explains why reactive electrophiles like sulforaphane and omaveloxolone have useful therapeutic windows, with efficacy at concentrations with few if any side effects. It is well established that the majority of NRF2 activators (in particular α,β unsaturated carbonyls and isothiocyanates) preferentially react with C151 out of the 27 cysteines in KEAP1. It is also recognized that some mechanism must chemically favor Class 1 electrophiles’ reaction with this sensor cysteine, given the much higher concentration of cellular thiols compared to C151. KEAP1 is present at ∼1 μM in the cytoplasm [33]. While a lower p*K*_a_ increases the percentage of this cysteine available to react with an electrophile, another mechanism is required to favor this reaction in the cellular milieu. For example, most cell types have 1–2 mM glutathione in the cytoplasm, with up to 10 mM in hepatocytes, and even under oxidative stress conditions the ratio of GSH/GSSG remains very high [41]. While the p*K*_a_ of the glutathione cysteine (∼8.6) limits its thiolate concentration to 60–120 μM at physiological pH, it is still much higher than the ∼0.8 μM C151 thiolate, with a p*K*_a_ of 6.9. In addition, glutathione-*S*-transferase enzymes catalyze the reaction of electrophiles with glutathione, including by lowering the p*K*_a_ to ∼6.6 [42]. The ability of therapeutic Class 1 NRF2 activator-electrophiles to react with KEAP1 C151 much more rapidly than with other cellular cysteines provides an explanation for their observed high selectivity for C151, and their potency and correlated low toxicity in cells, resulting in wide therapeutic windows for the most potent activators [36].

4D docking provides a model of pre-covalent binding of a Class 1 electrophile to the C151 active site. BTB X-ray crystal structures, while useful, represent the product of the addition reaction, rather than the pre-covalent ES complex. Using a 7-structure 4D model docked to DMF, *R*–SFN, *S*-SFN, omaveloxolone and CDDO-Me, several features of the active site stand out. Hydrophobic interactions appear to significantly contribute to catalysis, shown by the correlation of the hydrophobic interaction score for these NRF2 activators with both their ability to compete with mBBr for C151 and with their potency in keratinocytes. Omaveloxolone, the first designed FDA-approved NRF2 activator, utilizes 12 distinct residues within the active site for hydrophobic interactions. Of these 12, H129, K131, V132, and H154 make hydrophobic contributions to all four Class 1 electrophiles, and like all residues in the C151 pocket, are highly conserved [8]. In addition, each compound has a single hydrogen bond with a residue in the pocket, which appears to help orient the molecules, positioning their electrophilic carbons within ∼3–5 Å of C151. In studies that have examined CDDO and CDDO-like compound specificity using protein crystallography and structure-activity relationships, a hydrogen bond between the G148 backbone nitrogen and the oxygen of the α,β unsaturated carbonyl is present in each structure [24,43]. The positions of CDDO-Me and omaveloxolone in the docking model are similar to that of CDDO in the crystal structure, and a single hydrogen bond to G148 for both places the electrophilic carbon 3.2 Å from the thiolate. Interestingly, both *R*- and *S*-SFN also have a single hydrogen bond to G148 in the active site, orienting their electrophilic carbons 5.2 Å from the thiolate, and for DMF, G148 and R135 play a similar role. An enzyme brings relevant reactive groups within 3–6 Å of each other, holding them in position by restricting translational and rotational freedom, and effectively increases their local concentration, generally known as a proximity effect. The probability of a successful reaction and thus the rate of reaction are thereby significantly increased. In addition, at these distances, van der Waals attractive forces are maximized and repulsive forces are minimized, and sulfur, being more polarizable than oxygen or nitrogen, will have stronger attractive dispersion forces with a nearby atom. Finally, the G148 backbone nitrogen's hydrogen bond is positioned such that it could drive catalysis for each of the α,β unsaturated carbonyl compounds both by increasing the electrophilicity of the carbon atom and by stabilizing the negative charge that builds up on the oxygen in the transition state and the intermediate upon addition.

The need for a hydrogen bond to the carbonyl oxygen of an α,β unsaturated carbonyl is further supported by the results with non-Class 1 electrophiles 15d-PGJ_2_ and PGA_2_. Both compounds fit well into the hydrophobic pocket, with hydrophobic interaction scores that lie between those of omaveloxolone and the sulforaphane isomers, and both adopt a docking pose that places an electrophilic carbon within a reasonable reaction distance of the thiolate. However, the PGA_2_ poses lack any residue that could hydrogen bond with the carbonyl oxygen, and correspondingly, PGA_2_ is unable to compete with mBBr for C151. In contrast, pose 2 for 15d-PGJ_2_ positions the nitrogen of H154 an ideal 3.1 Å from the carbonyl oxygen, presumably to serve the role that the G148 backbone nitrogen does for the C151-targetted electrophiles. Correspondingly, the C151 active site is able to catalyze the addition of 15d-PGJ2, at least to some extent, shown by the mBBr competition assay. (We note that in this model, the δ-carbon of 15d-PGJ_2_ in the alkyl chain would react with C151, and not the endocyclic β-carbon that is more readily modified by glutathione [40], a prediction that remains to be tested.)

The ability of both Cd^2+^ and 15d-PGJ_2_ to block mBBr binding to C151 to some extent is of note, as these are both broadly considered to be independent of C151. Interestingly, the mouse embryonic KEAP1 C151S cell line used by the Yamamoto group to establish the five classes of NRF2 activators is hyperresponsive to CdCl_2_, an observation that to date is unexplained [19]. Our data suggest that Cd^2+^ reacts with C151 in WT KEAP1, but that unlike other modifications of this residue, this does not induce a conformational change sufficient to shut down NRF2 ubiquitination. Using a series of amino acid mutations at residue 151, we showed previously that NRF2 ubiquitination and subsequent antioxidant response element activation are dependent on the partial molar volume at this residue [44]. Thus, as a single ion, the partial molar volume of Cd^2+^ in complex with the C151 thiolate would likely lie well below that required to prevent NRF2 ubiquitination. Indeed, if C151 is more Cd^2+^-reactive than other sensor cysteines, this would lead to the observed hyperresponsive phenotype of the C151S mutant, with more Cd^2+^ available to react with its Class 4 sensor cysteines. In contrast, for 15d-PGJ_2_, the NRF2 response is the same in the WT and KEAP1 C151S mouse embryonic cell lines [10], supporting the idea that 15d-PGJ_2_ reacts faster with other KEAP1 cysteines. Regarding our results, the ability of 15d-PGJ_2_ to react with C151 within the context of the isolated BTB domain doesn't preclude its potential ability to react even faster with other KEAP1 cysteines. There is evidence from mass spectrometry experiments that 15d-PGJ_2_ prefers to react with multiple other cysteines over C151, with C288 among those preferentially labeled for mouse, but not human, KEAP1 [45,46].

The p*K*_a_ of 6.9 for C151 is optimized for reactivity. While only the thiolate, and not the protonated thiol, is a nucleophile, this factor will only be important until the p*K*_a_ drops below the pH of the solution. Extensive experiments in the late 1970s into the 1980s established that the rate constant for thiolate with a disulfide decreases with decreasing p*K*_a_, as the factors which lower a p*K*_a_ also stabilize the negatively charged thiolate, reducing its nucleophilicity (Fig. 3C, summarized in Ref. [47]). Thus, the rate constant for a thiol group is expected to be optimal when the p*K*_a_ is near the experimental pH. The determined p*K*_a_ for C151 of 6.9 is likely then near optimal in the cytoplasm, where KEAP1 is located.

The understanding that an active site around C151 drives the kinetics of addition beyond optimizing p*K*_a_ is useful for the design/discovery and development of NRF2-activating therapeutics. For example, there are conflicting data in the literature as to whether the *R*–SFN stereoisomer is a more potent/effective NRF2 activator than the *S*-SFN isomer [48], or whether they are in fact equivalent NRF2 activators [49]. (While *R*–SFN is often stated to be the naturally occurring form, the amount of *S*-SFN in preparations of broccoli parts can be quite high, up to ∼40 % in broccoli sprout stems [50].) Their equal rates of reaction with BTB-C151, in competition with mBBr, indicates that any differences observed between the stereoisomers in a particular cell type are likely to due to factors other than their ability to free NRF2 from KEAP1 repression. Whatever these factors are for a given cell type, the affinity of the C151 active site for an electrophile and its ability to orient the electrophilic carbon to C151 and thus catalyze the reaction are likely critical factors for discovery and prediction of potent activators. Accordingly, an important implication for screening efforts to discover Class 1 NRF2 activators with high specificity is that affinity for the C151 pocket alone will likely not correlate with the rate of reaction of an electrophile with C151. Rather, *in silico* screening methods to isolate predicted hits will likely require the sophistication of screening for orientation of the molecule in the pocket, taking into account the various factors important for catalysis.

## 4 Methods

### 4.1 KEAP1 BTB domain purification

cDNA encoding the human KEAP1 BTB domain (residues 48–180, including an S172A point mutation [24]) was codon-optimized and cloned into the pSKB3 vector, incorporating a TEV protease-cleavable 6x-His tag. In this study, the descriptor WT is applied to protein that contains only the S172A mutation, and C151S contains the S172A mutation in addition to the indicated point mutation. Protein expression was induced in Rosetta (DE3) pLysS cells with 0.2 mM IPTG at 18 °C for 18 h. Protein was purified from cell lysates by nickel affinity chromatography to ∼98 % purity. The His tag was cleaved with TEV protease, and the protease and cleaved 6x-His tag were captured via incubation with Ni-NTA resin. The protein was stored at −80 °C in aliquots in 25 mM Tris, 1 mM TCEP, 10 % glycerol, pH 8.

### 4.2 Monobromobimane modification of BTB-C151 and N-acetyl cysteine

Single-use aliquots of 10 mM mBBr in acetonitrile were stored at −80 °C. In all experiments with the KEAP1 BTB domain protein, the protein was reduced with 1 mM DTT at room temperature for 1 h and subsequently buffer exchanged into 15 mM Tris, pH 8 using Zeba 7 kDa MWCO Spin Desalting Columns. The 1 mM DTT concentration is high enough to reduce C151 but low enough to be completely removed by desalting. In fluorescence experiments, incomplete removal of DTT results in slow formation of a DTT-monobimane conjugate, producing fluorescence independent of C151. To ensure DTT removal, desalting volumes were limited to 35 μL and/or two desalting steps were performed. In addition, the BTB protein was used as soon as possible after desalting, as C151 rapidly re-oxidizes after DTT removal. Reactions were carried out at 25 °C under low light conditions in 15 mM acetate/15 mM 2-(*N*-morpholino)ethanesulfonic acid/30 mM Tris reaction buffer [28] (to obtain pHs from 4 to 9) in a black 96 well plate with 100 μL reaction volumes. Fluorescence detection was performed on a BMG Labtech ClarioStar microplate reader with Ex/Em of 396/482 nm. Experiments using NAC were performed in 10 mM CAPS buffer, pH 11 and were otherwise identical to experiments using KEAP1 BTB.

### 4.3 p*K*_a_ determination

For the p*K*_a_ determination of C151, 0.8 μM WT KEAP1 BTB domain was reacted with 16 μM mBBr in reaction buffer at every half pH unit from pH 4 to pH 9 in triplicate. Reactions were initiated with mBBr, and the fluorescence was measured immediately after mixing. The RFU at each pH were plotted in GraphPad Prism, and the data were fit to a log(inhibitor) vs. response 4-parameter variable slope non-linear regression. The IC_50_ value determined by the fit was taken as the p*K*_a_, and three replicate runs were averaged to obtain a value with standard deviation.

### 4.4 Direct competition assay

To test the direct competition between select electrophiles and mBBr for reactivity with WT KEAP1 BTB domain, protein was reacted simultaneously with an electrophile and mBBr. Reactions were initiated by adding 10 μL of pre-mixed electrophile (or DMSO control) and mBBr to wells containing 90 μL of WT BTB in reaction buffer, and readings were done as for mBBr reactions with BTB alone. Final concentrations were 0.8 μM WT KEAP1 BTB domain, 8 μM electrophile (or 1 % DMSO), and 8 μM mBBr.

### 4.5 Intact mass spectrometry

The KEAP1 BTB domain (WT or C151S) was reacted with mBBr at pH 8, with final concentrations of 1.6 μM and 16 μM, respectively. The reaction was monitored by fluorescence to ensure reaction and subsequently buffer exchanged into 15 mM Tris, pH 8 using Zeba 7 kDa MWCO spin desalting columns to remove excess mBBr. LC-MS experiments were performed on a SCIEX Exion HPLC coupled to a SCIEX 5600+ TripleTOF (Q-TOF, quadrupole time-of-flight) tandem mass spectrometer. The SCIEX DuoSpray ion source was used, with the electrospray (ESI) probe set to positive polarity mode. The data acquisition mode was set to TOF-MS. The source and gas parameters were set as follows: declustering potential = 100 V, collision energy = 10 V, ion source gas 1 = 50, ion source gas 2 = 50, curtain gas = 25, temperature = 500 °C, ionspray voltage = 5500. The scan range was 300–3000 *m*/*z*. Injection volumes were 20 μL. Chromatography was performed on an Agilent BioHPLC PLRP-S reversed-phase PS-divinylbenzene column (1000 Å, 5 μm, 50 × 2.1 mm), and the column temperature was set to 40 °C. Mobile phase A was HPLC-grade water with 0.1 % formic acid, and mobile phase B was acetonitrile with 0.1 % formic acid at a flow rate of 0.4 mL/min. The LC gradient was as follows: 5-min hold with 100 % A, isocratic ramp to 95 % B (6 min), hold at 95 % B (6–11 min), isocratic ramp back to 100 % A (12 min) and hold at 100 % A (12–14 min). Data analysis was performed on SCIEX PeakView software. Protein molecular weight reconstruction was done with the following reconstruction settings: input mass range: start *m*/*z* = 750 to stop *m*/*z* = 1,800, output mass range: 15,000 Da to 16,000 Da, step mass of 1.0 Da, isotope resolution set to ‘resolved,’ and the charge agent set to ‘H^+^.’

### 4.6 Docking electrophiles using the 4D-flexible model of the KEAP1 BTB domain

Seven BTB structures served as a template for the development of a 4D model of a flexible receptor, one apo structure and six with electrophiles covalently bound to C151 (PDB IDs 4cxi (apo) and 4cxt (CDDO) [24], 6ffm (a 3-bromo-4,5-dihydroisoxazole derivative) [51], 5dad (TX64014) and 5daf (TX64063) [43], 5git (britanin) [21], and 7x4x (monoethyl fumarate) [52]. Using the icmpocketfinder program, a ligand-binding pocket around the target residue C151 was found, and a docking box was generated 5 Å around the identified pocket (*SI Appendix*, Fig. S2). Using Monte Carlo simulation, ICM simultaneously perturbed the ligand's position, orientation, and internal torsions while allowing the side-chain rotamers that line the pocket to adjust. Each trial move was followed by local energy minimization. The binding scores were calculated using version 2016 of the ICM docking scoring tool. The conformation with the lowest binding energy out of 1000 trials was recorded as the most probable binding configuration for the selected electrophile. The ligand-protein docking program was run on a workstation of CPU (AMD Ryzen Threadripper 7970X), GPU (Nvidia GeForce RTX 3080Ti), and 512G memory. The 2D interaction diagrams generated by ICM were recreated in Biorender for increased resolution [53].

### 4.7 Crystallization, X-ray data collection, and structure determination

For crystallization, 1 mM BTB (15.2 mg/mL) protein in 1 mM TCEP was reacted with 4 mM mBBr (dissolved in DMSO) such that the final reaction contained 1.1 % DMSO. The reaction was quenched and subsequently purified by size-exclusion chromatography (Superdex S200), followed by concentration to 11.5 mg/mL in 25 mM Tris, 10 % glycerol, 1 mM TCEP, pH 8. Monobimane was covalently bound to 94 % of the BTB protein as assessed by mass spectrometry.

Crystallization was adapted from previously reported conditions [24] using hanging-drop vapor diffusion with a 1:1 vol ratio of protein to precipitant. Optimized crystals grew at 16 °C with precipitant solution containing 0.2 M lithium acetate, 5 % glycerol, and 14–20 % PEG 3350. Crystals were cryoprotected with crystallization solution supplemented with 20 % glycerol, and flash-cooled in liquid nitrogen.

X-ray diffraction data collected at beamline 17-ID-1 (AMX) of the National Synchrotron Light Source II were indexed, merged, and scaled using XDS [54]. Like previously reported BTB structures, the crystal belonged to space group *P*6_5_22 with one molecule in the asymmetric unit and diffracted to 1.80 Å resolution. The structure was determined by molecular replacement in Phaser [55] in the Phenix suite [56] using the apo form of the BTB domain of KEAP1 (PDB 4cxi) as a search model. Iterative rounds of model building in Coot [57] and reciprocal space refinement in phenix.refine [58] were performed. Electron density maps near C151 indicated that the ligand was present, but the density was discontinuous and inconsistent with any single conformation. In the final model, two conformations of cysteine-bound monobimane were chosen to be modeled and refined. The ligand and C151 occupancies for each conformation were set to 50 % during model building that were then refined to 52 % and 48 %. Atomic coordinates and structure factors were deposited with the Protein Data Bank (PDB) with the PDB ID 9phr. Images of structures and electron density maps were rendered using PyMOL [59].

## CRediT authorship contribution statement

**Matthew R. Schnell:** Formal analysis, Investigation, Writing – original draft, Writing – review & editing. **Tianhua Zhai:** Formal analysis, Investigation, Writing – original draft, Writing – review & editing. **Edwin R. Ragwan:** Formal analysis, Investigation, Visualization, Writing – original draft, Writing – review & editing. **Hannah Jung:** Investigation, Writing – review & editing. **Jiayu Zhang:** Formal analysis, Investigation, Writing – review & editing. **Anthony F. Lagalante:** Formal analysis, Methodology, Visualization. **Yan Kung:** Formal analysis, Investigation, Validation, Writing – original draft, Writing – review & editing. **Daniel A. Kraut:** Conceptualization, Formal analysis, Writing – original draft, Writing – review & editing. **Zuyi Huang:** Data curation, Formal analysis, Investigation, Writing – original draft, Writing – review & editing. **Aimee L. Eggler:** Conceptualization, Data curation, Formal analysis, Funding acquisition, Investigation, Project administration, Visualization, Writing – original draft, Writing – review & editing.

## Declaration of competing interest

The authors declare that they have no known competing financial interests or personal relationships that could have appeared to influence the work reported in this paper.

## Data Availability

Data will be made available on request.
