# Corrigendum to “KEAP1 C151 active site catalysis drives electrophilic signaling to upregulate cytoprotective enzyme expression” [Redox Biology 88 (2025) 103906]

**DOI:** 10.1016/j.redox.2025.104000

**Published:** 2025-12-30

**Authors:** Matthew R. Schnell, Tianhua Zhai, Edwin R. Ragwan, Hannah Jung, Jiayu Zhang, Anthony F. Lagalante, Yan Kung, Daniel A. Kraut, Zuyi Huang, Aimee L. Eggler

**Affiliations:** a Department of Chemistry and Biochemistry, Villanova University, Villanova, PA, 19085, USA; b Department of Biochemistry and Biophysics, University of Pennsylvania, Philadelphia, PA, 19104, USA; c Department of Biostatistics, Epidemiology and Informatics, University of Pennsylvania Perelman School of Medicine, Philadelphia, PA, 19104, USA; d Department of Chemistry, Bryn Mawr College, 19010, USA; e Department of Chemical and Biological Engineering, Villanova University, Villanova, PA, 19085, USA

The authors regret that the following funding source was omitted from the Acknowledgments section of the original article:

“This work received funding from Villanova University's Falvey Memorial Library Scholarship Open Access Reserve (SOAR) Fund.”

The authors would like to apologise for any inconvenience caused.

