# Supplementary material for: KEAP1 C151 active site catalysis drives electrophilic signaling to upregulate cytoprotective enzyme expression
Source: Redox Biol. 2025 Oct 28;88:103906. doi: 10.1016/j.redox.2025.103906 (PMC12634842; doi:10.1016/j.redox.2025.103906)
Supplement: Multimedia component 1 [file mmc1.pdf]

Supplemental Information Appendix

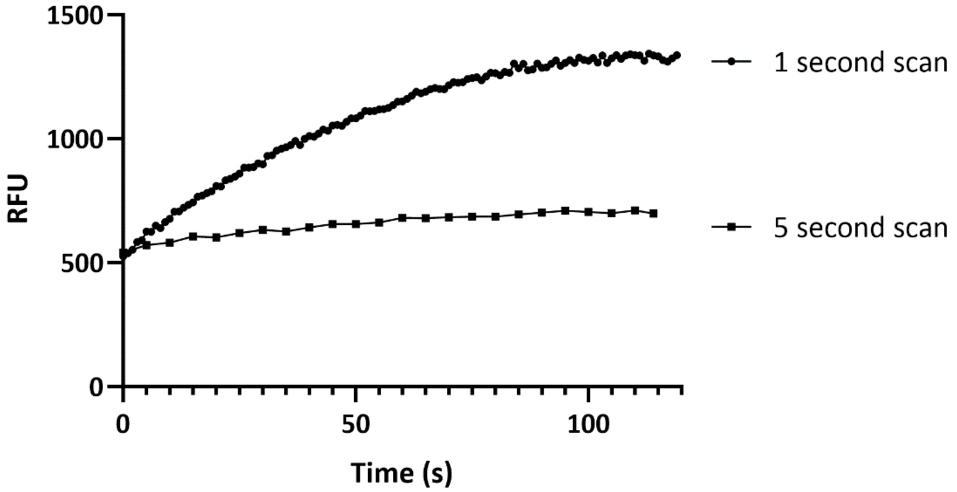

Supplemental Fig. S1. mBBr produces background fluorescence that increases in intensity with more frequent excitations. mBBr (8 μM) in pH 8 reaction buffer was scanned either once every second or once every 5 seconds for 2 minutes.

Supplemental Table S1: Data collection and refinement statistics

	Monobimane-bound BTB
PDB entry	9PHR
**Data Collection**	
Wavelength (Å)	0.9201
Resolution range (Å)	32.36 - 1.801 (1.94 - 1.8)
Space group	P 6_5 2 2
Unit cell dimensions	
a, b, c (Å)	42.976, 42.976, 266.628
α, β, γ (°)	90, 90, 120
Total reflections	532118 (96297)
Unique reflections	14489 (2786)
Multiplicity	36.7 (34.6)
Completeness (%)	99.07 (95.82)
Mean I/sigma(I)	12.45 (0.72)
Wilson B-factor (Å)	45.08
R_{merge}	0.1432 (4.02)
R_{meas}	0.1454 (4.079)
R_{pim}	0.02439 (0.6793)
$CC_{1/2}$	0.999 (0.485)
CC*	1 (0.808)
**Refinement**	
Reflections used in refinement	14393 (2702)
Reflections used for R-free	729 (146)
R_{work}	0.2339 (0.3541)
R_{free}	0.2820 (0.3817)
Number of non-hydrogen atoms	1062
macromolecules	1018
ligands	28
solvent	16
Protein residues	131
RMS for bonds (Å)	0.011
RMS fir angles (deg)	1.42
Ramachandran favored (%)	97.67
Ramachandran allowed (%)	2.33
Ramachandran outliers (%)	0
Rotamer outliers (%)	0.9
Average B-factor (Å^2)	68.38
macromolecules	68.73
ligands	65.26
solvent	51.68
Molprobity Clashscore	5.41

* Statistics for the highest resolution shell are shown in parentheses.

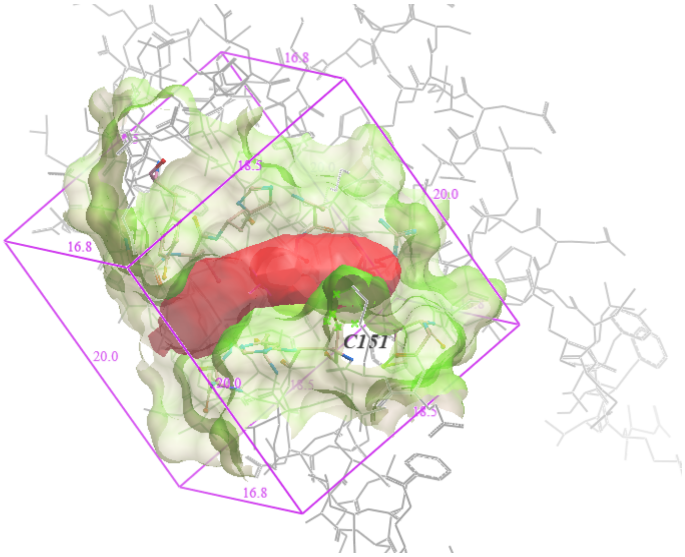

Supplemental Fig. S2. Docking box of KEAP1 receptor. The pocket (red) was found by Molsoft ICM icmpocketfinder tool with default tolerance (4.6), which covered the CDDO binding sites. Docking box was generated around the identified pocket, which was 18.5x16.8x20.0 $Å^3$.

Supplemental Table S2. Post- and pre-covalent ICM docking of mBBr and the BTB-C151 pocket

	Post-covalent		Pre-covalent	
Pose	Score	RTCNNscore	Score	RTCNNscore
1	-12	-16.054	-15.29	-19.333
2	-11.67	-11.597	-14.58	-17.474
3	-11.15	-15.296	-13.80	-21.266
4	-10.2	-15.372	-13.44	-20.962
5	-10.12	-14.690	-13.36	-21.500
6	-8.484	-14.384	-12.23	-19.003
7	-8.243	-11.567	-12.03	-18.934
8	-7.776	-14.496	-11.62	-18.186
9	-7.201	-13.220	-11.09	-18.214
10	-7.097	-16.252	-10.69	-18.017

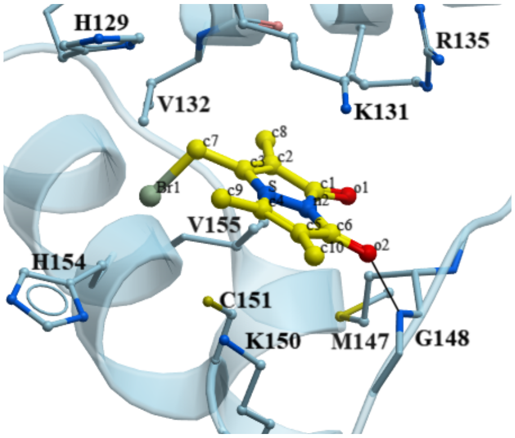

Supplemental Fig. S3. Pose 1 from the ICM 4D docking of mBBr to the BTB-C151 pocket. Numbering corresponds to Supplementary Table S3.

Supplemental Table S3. Pre-covalent ICM docking hydrogen bond and hydrophobic interactions of mBBr with the BTB-C151 pocket

	Pose 1			Pose 2			Pose 4			Pose 6		
	atom	size	distance	atom	size	distance	atom	size	distance	atom	size	distance
K131 [a]	—	—	—	—	—	—	o1	1.30	2.90	—	—	—
G148	o2	0.93	2.71	o2	1.02	2.88	—	—	—	—	—	—
H129	c7	1.26	3.68	c7	1.60	3.26	c10	2.54	3.24	Br1	1.15	3.97
K131	c7	2.63	3.70	c9	2.30	3.87	—	—	—	c5	2.42	3.93
V132	c8	1.83	3.10	c9	1.37	3.25	c9	1.58	3.17	c8	1.54	3.14
R135	c8	0.76	3.89	c10	0.84	3.68	Br1	0.49	3.91	—	—	—
M147	—	—	—	c10	1.42	3.00	—	—	—	c10	0.99	4.47
G148	—	—	—	—	—	—	—	—	—	c10	0.67	4.36
K150	c10	0.58	3.74	—	—	—	—	—	—	—	—	—
C151	c4	2.13	4.14	c5	2.07	4.23	c7	1.99	3.61	c3	1.76	4.18
H154	—	—	—	Br1	1.20	3.62	c9	1.01	3.37	c8	0.45	4.14
V155	—	—	—	c10	0.64	3.90	Br1	0.77	3.71	—	—	—

[a] Blue shading indicates a hydrogen bond interaction. Green shading indicates a hydrophobic interaction.

Supplemental Table S4. Top pre-covalent ICM docking poses for the indicated Class 1 electrophiles

	POSE	SCORE	NATOM	NFLEX	HBOND	HPHOB	VWINT	EINTL	DSOLV	SOLEL	MF SCORE	DTSSC
Omaveloxolone	**1**	-19.41	84	2	-4.24	-6.41	-23.8	5.18	10.83	10.55	-73.03	2.37
	2	-7.27	84	2	-1.67	-5.65	-13.4	2.87	9.78	5.89	-83.20	2.67
	3	-6.87	84	2	-1.91	-5.79	-15.8	4.47	11.15	8.10	-57.11	1.80
	4	-6.22	84	2	-1.05	-5.57	-17.4	4.83	10.95	7.93	-97.49	2.47
	5	-5.67	84	2	-0.80	-5.84	-13.6	3.74	8.68	5.91	-66.69	2.34
	6	-5.27	84	2	-0.76	-5.92	-18.2	5.35	9.62	10.82	-73.61	2.31
	7	-4.26	84	2	-0.16	-6.36	-18.6	3.91	10.52	11.45	-88.32	2.35
	8	-3.85	84	2	-1.91	-6.19	-16.0	4.87	11.98	12.18	-91.99	2.49
	9	-3.69	84	2	-0.65	-5.90	-12.7	3.67	10.08	5.53	-34.64	1.72
	10	2.90	84	2	-0.63	-6.16	-11.5	5.14	12.39	9.87	-91.66	3.09
CDDO-Me	1	-14.92	80	2	-2.47	-6.36	-21.14	2.17	10.72	9.36	-81.14	2.33
	2	-12.52	80	2	-4.77	-5.49	-9.72	1.20	8.52	7.00	-66.04	2.35
	3	-3.91	80	2	-1.39	-6.36	-17.85	5.83	13.32	10.63	-87.53	2.33
	4	-1.96	80	2	0.00	-6.28	-12.64	5.70	7.45	7.10	-74.29	2.02
	5	-1.75	80	2	-1.98	-4.62	-13.13	4.94	13.76	7.58	-71.70	2.79
	6	-1.29	80	2	-0.07	-5.81	-14.34	1.96	11.13	9.69	-92.06	2.29
	7	-0.19	80	2	-1.87	-5.63	-13.90	3.69	14.39	12.14	-88.12	3.33
	8	1.04	80	2	-1.44	-4.75	-9.61	3.55	13.47	6.11	-85.24	2.46
	9	2.22	80	2	-0.78	-6.08	-10.84	5.90	12.68	7.38	-85.04	2.99
	10	9.30	80	2	-0.25	-6.09	-7.48	6.51	13.79	9.14	-78.78	2.23
R-SFN	1	-13.26	21	5	-3.84	-3.79	-13.0	2.92	6.82	3.22	-21.91	1.66
	2	-8.56	21	5	-0.69	-3.83	-14.6	1.72	4.05	5.04	-39.81	1.20
	3	-7.60	21	5	-0.84	-3.94	-14.9	1.67	6.25	5.05	-32.93	1.51
	4	-7.37	21	5	-0.33	-3.87	-14.2	0.89	4.98	4.55	-39.90	1.22
	5	-6.70	21	5	-1.88	-3.49	-11.7	1.31	6.60	5.06	-15.40	1.15
	6	-5.81	21	5	0.00	-3.60	-15.0	2.18	5.26	5.12	-41.72	1.28
	7	-5.11	21	5	0.00	-3.53	-14.1	1.50	5.43	5.07	-42.14	1.38
	8	-5.00	21	5	0.00	-3.74	-13.6	1.39	5.77	4.40	-23.72	1.23
	9	-4.53	21	5	-0.65	-3.51	-10.8	4.36	4.78	1.70	-1.02	0.72
	10	-2.95	21	5	-0.96	-3.86	-12.4	4.70	6.04	6.20	-17.27	1.30
S-SFN	1	-11.35	21	5	-1.09	-3.86	-15.74	0.62	5.13	3.80	-26.52	1.14
	2	-8.98	21	5	-0.85	-3.94	-15.71	0.77	5.39	6.09	-29.78	1.39
	3	-6.88	21	5	-0.72	-3.36	-11.67	1.25	4.95	2.12	-5.12	0.76
	4	-6.12	21	5	-1.06	-3.04	-13.97	0.91	6.64	6.11	-41.11	0.75
	5	-6.03	21	5	-2.13	-3.70	-10.85	1.45	7.14	5.24	-42.25	0.88
	6	-5.99	21	5	-1.56	-3.85	-11.67	1.74	6.15	5.39	-37.99	1.12
	7	-5.75	21	5	0.00	-3.46	-14.62	3.09	4.55	4.45	-45.44	1.11
	8	-5.08	21	5	-1.30	-3.47	-13.25	2.84	6.69	6.13	-25.20	1.30
	9	-5.03	21	5	0.00	-3.60	-14.29	2.88	4.50	5.39	-32.11	1.24
DMF	1	-13.87	18	0	-2.47	-2.63	-16.2	1.08	8.96	4.45	-13.57	0.81
	2	-12.90	18	0	-3.24	-2.33	-11.8	1.53	9.73	0.59	-41.09	0.62
	3	-12.65	18	0	-2.59	-2.42	-10.7	2.12	6.19	0.52	0.87	0.72
	4	-12.01	18	0	-1.50	-2.99	-13.2	0.17	7.02	2.46	-45.53	1.01
	5	-11.57	18	0	-2.46	-2.65	-10.4	0.23	8.86	0.07	-34.03	0.82
	6	-11.50	18	0	-0.59	-3.29	-16.4	1.20	7.15	3.76	-38.39	0.87
	7	-11.48	18	0	-2.24	-2.83	-13.4	0.88	9.28	3.07	-18.22	1.24
	8	-10.91	18	0	-2.47	-2.55	-13.9	2.10	10.02	3.33	-19.33	0.51
	9	-10.38	18	0	-1.40	-2.76	-11.9	0.06	6.61	2.89	-28.92	1.38
	10	-9.95	18	0	-1.80	-2.82	-10.9	0.00	7.77	2.38	-19.56	0.42

Supplemental Table S5. Pre-covalent ICM docking hydrogen bond and hydrophobic interactions for CDDO-Me and Omaveloxolone with the C151 pocket

	CDDO-Me			Omaveloxolone		
	atom	size	distance	atom	size	distance
G148[a]	o1	1.41	2.80	o2	1.45	2.81
Y85	c32	1.23	3.94	c7	1.27	4.15
Q86	—	—	—	c33	0.30	4.31
A88	c32	0.41	4.37	c33	0.66	4.22
H129	c19	2.44	3.37	f2	2.61	3.41
K131	c8	1.80	3.17	c15	1.77	3.19
V132	c16	0.59	4.04	c10	0.57	4.05
R135	c10	1.73	3.19	c28	1.77	3.20
M147	c11	0.95	4.02	c29	0.97	4.06
K150	c3	1.20	3.90	c32	1.19	3.96
C151	c2	2.42	3.81	c27	2.45	3.83
H154	c30	1.34	3.50	c24	1.46	3.40
V155	c11	0.28	4.29	c29	0.27	4.31
Interaction Map	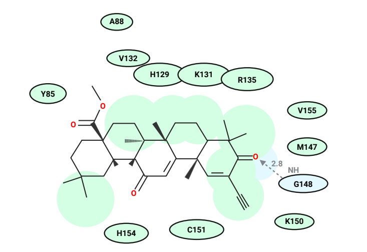			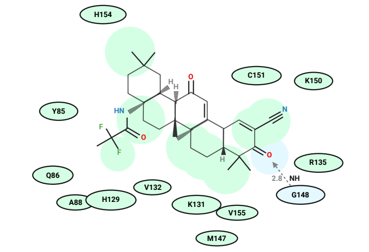		
Atom Numbers/ Molecule Orientation	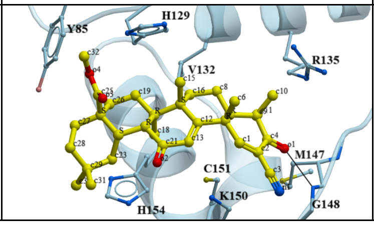			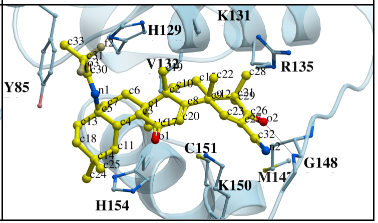		

[a] Blue shading indicates a hydrogen bond interaction. Green shading indicates a hydrophobic interaction.

Supplemental Table S6. Pre-covalent ICM docking hydrogen bond and hydrophobic interactions for *S*-SFN, *R*-SFN and the two DMF poses with the C151 pocket

	S-SFN			*R*-SFN			DMF Pose 1			DMF Pose 7		
	atom	size	distance	atom	size	distance	atom	size	distance	atom	size	distance
R135[a]	—	—	—	—	—	—	o1	1.40	2.97	—	—	—
G148	o1	0.64	2.76	o1	0.90	3.07	—	—	—	o2	0.76	2.95
H129	c6	2.33	3.49	c6	2.60	3.41	—	—	—	c6	1.44	3.48
K131	c3	1.69	4.00	c1	1.59	3.96	c3	1.73	3.89	c3	1.73	3.86
V132	c5	1.27	3.37	c4	1.00	3.20	c6	1.14	3.30	—	—	—
R135	—	—	—	c1	1.14	3.84	—	—	—	—	—	—
M147	—	—	—	c1	1.15	3.71	c2	1.94	3.71	—	—	—
C151	c3	1.32	3.99	c3	1.28	3.87	c6	0.91	4.29	c4	1.77	3.47
H154	c6	1.46	4.26	c5	1.08	3.66	c6	0.37	4.14	—	—	—

Interaction Map

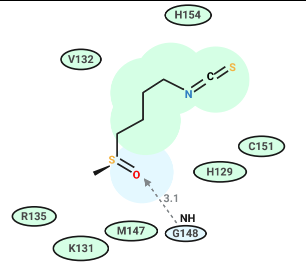

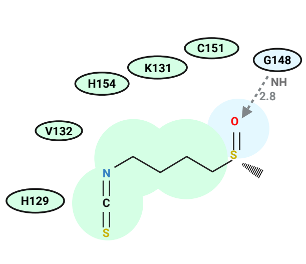

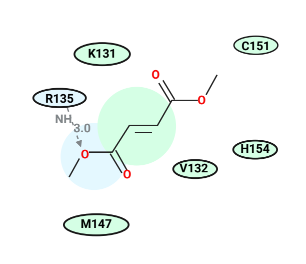

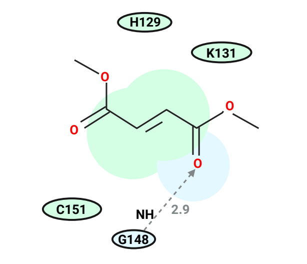

Atom Numbers/ Molecule Orientation

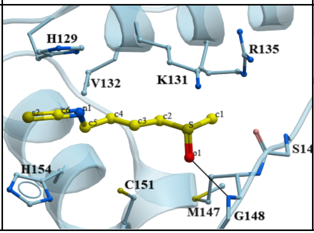

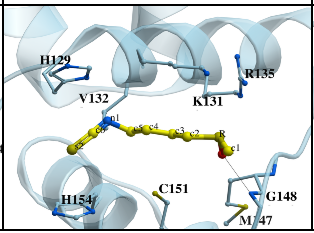

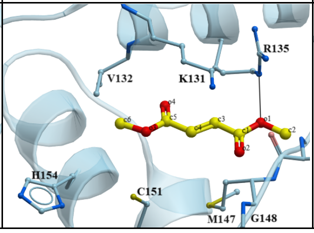

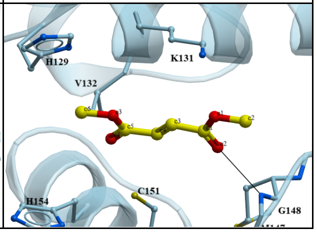

[a] Blue shading indicates a hydrogen bond interaction. Green shading indicates a hydrophobic interaction.

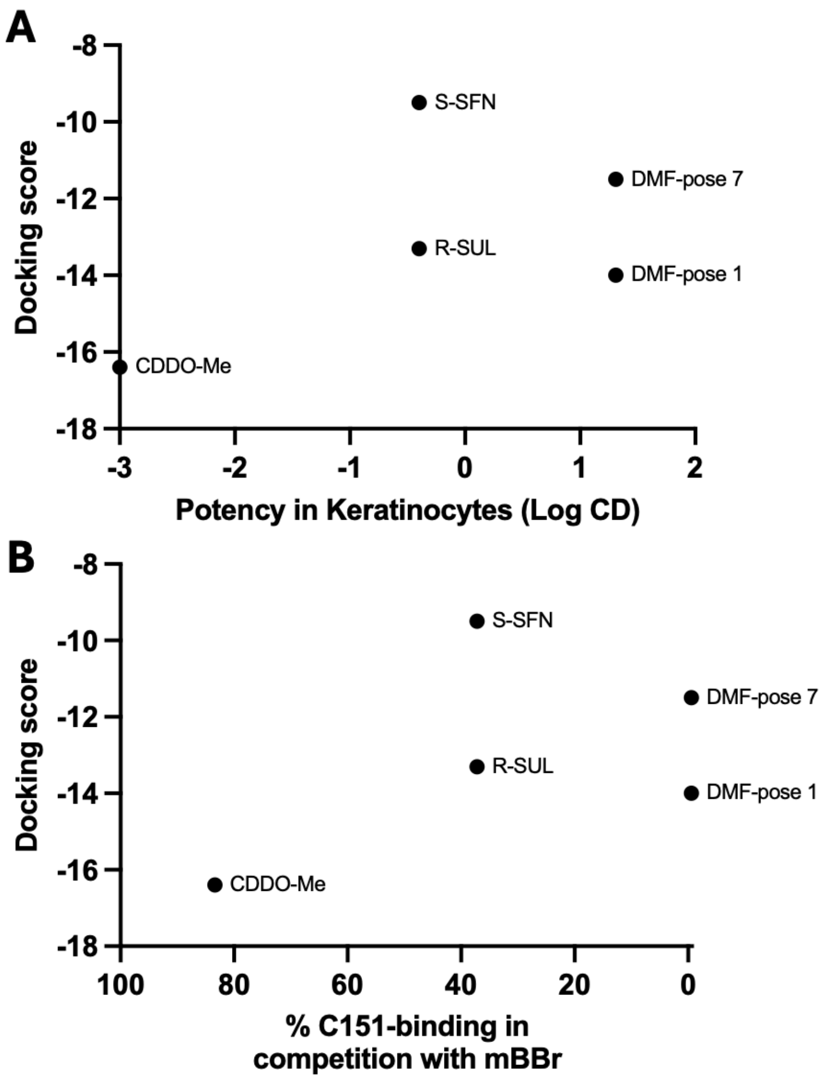

Supplemental Fig. S4. Plotting the docking scores of electrophiles shows no direct correlation with potency in keratinocytes *(A)* or ability to compete with mBBr for C151 *(B)*.

Supplemental Table S7. Top pre-covalent ICM docking poses for the indicated non-Class 1 electrophiles

	POSE	SCORE	NATOM	NFLEX	HBOND	HPHOB	VWINT	EINTL	DSOLV	SOLEL	MF SCORE
15d-PGJ$_2$	1	-8.17	51	12	-1.44	-6.23	-26.33	12.36	7.58	8.61	-85.51
	2	-7.61	51	12	-2.58	-5.33	-23.94	7.98	11.03	9.34	-103.31
	3	-7.16	51	12	-2.27	-6.27	-24.29	11.88	9.01	9.08	-94.26
	4	-6.26	51	12	-0.64	-6.69	-26.00	8.15	8.15	11.56	-74.93
	5	-5.83	51	12	-0.20	-6.37	-26.13	9.09	8.82	8.76	-82.06
	6	-3.92	51	12	0.00	-6.14	-25.25	7.18	11.41	8.16	-99.28
	7	-3.78	51	12	-0.39	-6.79	-19.64	8.01	6.47	6.89	-68.88
	8	-3.30	51	12	-1.88	-6.09	-22.91	9.45	11.28	10.78	-84.31
	9	-2.82	51	12	-2.07	-5.71	-23.12	9.68	11.23	12.02	-83.09
	10	-1.76	51	12	-0.13	-5.89	-22.69	9.28	9.66	7.86	-65.17
PGA$_2$	1	-10.80	54	14	-5.96	-5.25	-27.08	9.81	16.68	7.57	-104.98
	2	-9.82	54	14	-2.84	-6.09	-22.91	8.80	7.96	7.12	-100.57
	3	-9.22	54	14	-1.71	-5.58	-24.89	4.37	10.23	7.66	-124.52
	4	-8.59	54	14	-3.69	-5.45	-26.81	9.32	12.82	11.52	-77.98
	5	-6.28	54	14	-2.93	-5.87	-22.35	6.69	11.48	9.70	-133.32
	6	-6.87	54	14	-3.22	-5.48	-24.33	8.49	12.14	10.12	-80.42
	7	-5.25	54	14	-3.55	-5.58	-22.25	9.20	14.36	7.63	-98.28
	8	-2.62	54	14	-1.87	-5.72	-23.24	8.85	12.27	9.53	-98.42
	9	-1.04	54	14	-0.08	-5.76	-26.78	12.04	11.29	8.64	-75.30
	10	1.67	54	14	-0.62	-5.98	-21.75	7.04	13.08	9.95	-83.87
